# Counting the number of enzymes immobilized onto a nanoparticle-coated electrode

**DOI:** 10.1007/s00216-017-0829-1

**Published:** 2017-12-27

**Authors:** Jenny Bergman, Yuanmo Wang, Joakim Wigström, Ann-Sofie Cans

**Affiliations:** 1 Department of Chemistry and Molecular Biology, Gothenburg University, 41296 Gothenburg, Sweden; 2 Department of Chemistry and Chemical Engineering, Chalmers University of Technology, 41296 Gothenburg, Sweden

**Keywords:** Gold nanoparticles, Immobilized enzyme, Enzyme quantification, Microelectrode, Electrochemical stripping, Glucose oxidase

## Abstract

To immobilize enzymes at the surface of a nanoparticle-based electrochemical sensor is a common method to construct biosensors for non-electroactive analytes. Studying the interactions between the enzymes and nanoparticle support is of great importance in optimizing the conditions for biosensor design. This can be achieved by using a combination of analytical methods to carefully characterize the enzyme nanoparticle coating at the sensor surface while studying the optimal conditions for enzyme immobilization. From this analytical approach, it was found that controlling the enzyme coverage to a monolayer was a key factor to significantly improve the temporal resolution of biosensors. However, these characterization methods involve both tedious methodologies and working with toxic cyanide solutions. Here we introduce a new analytical method that allows direct quantification of the number of immobilized enzymes (glucose oxidase) at the surface of a gold nanoparticle coated glassy carbon electrode. This was achieved by exploiting an electrochemical stripping method for the direct quantification of the density and size of gold nanoparticles coating the electrode surface and combining this information with quantification of fluorophore-labeled enzymes bound to the sensor surface after stripping off their nanoparticle support. This method is both significantly much faster compared to previously reported methods and with the advantage that this method presented is non-toxic.

**Graphical abstract:**
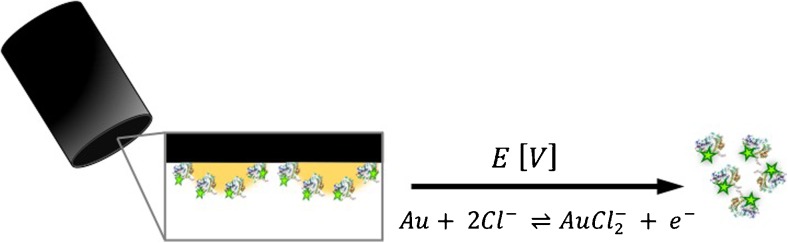
A new analytical method for direct quantification of the number of enzymes immobilized at the surface of gold nanoparticles covering a glassy carbon electrode using anodic stripping and fluorimetry

A new analytical method for direct quantification of the number of enzymes immobilized at the surface of gold nanoparticles covering a glassy carbon electrode using anodic stripping and fluorimetry

## Introduction

Since Clark and Lyons developed the first enzyme-based biosensor for monitoring glucose in 1962 [1], there have been a tremendous variety of enzyme-based biosensors created for different applications such as food industry, pharmacology, medicine, environmental analysis, and chemistry [2–7]. Electrochemical detection of enzymatic product formation is a widely used approach in many biosensor systems to indirectly probe an analyte serving as the enzyme substrate and that is not electroactive and therefore not directly detectable using electrochemistry [8–11]. Common electrode materials for constructing enzymatic sensors include carbon and different noble metals, e.g., platinum and gold. It has previously been shown that immobilizing enzyme on a high curvature surface is beneficial to retain enzymatic activity by minimizing changes in tertiary structure of the enzyme upon attachment and preventing denaturation [12, 13]. In order to achieve high curvature topology at the electrode for enzymes to bind, surfaces can be modified with various nanomaterials such as nanotubes and nanoparticles (NPs) [14]. Additionally the immobilization of nanostructures will increase the surface area of the electrode and thereby allow a higher enzyme loading. The approach of modifying electrode surfaces with metal nanoparticles has been extensively used [15–18]. Gold is a material often exploited, due to its high biocompatibility and its enhanced activity to oxidize/reduce electroactive reporter molecules, e.g. hydrogen peroxide from enzymatic reactions involving the analyte [19, 20]. The nanoparticles can be deposited onto the electrode surface by several methods including drop casting a solution of nanoparticles [21, 22], seed-mediated growth method [16, 23], or electrodeposition from a gold ion containing solution [24, 25]. For biosensor applications in biological systems, the most essential parameters to optimize are selectivity and sensitivity due to the challenge of detecting just the analyte when probing in a complex biological matrix and to ensure analytes present at physiological very low concentration are detectable. In addition, for certain applications, sensor temporal resolution needs to be fast enough for detection of biologically relevant fluctuations of analyte on the time scale that these events occur. The sensor size needs to be considered for optimizing desired spatial resolution and for in vivo probes to minimize tissue damage upon insertion. Deeper investigations of how enzymes interact with the electrode surface upon immobilization are of great importance when constructing a biosensor and in optimizing the sensor performance to meet the properties needed for the sensor application. Our research has been directed towards the development of biosensors for analyzing rapid release of non-electroactive neurotransmitters from secretory cells. We recently showed that a thin enzyme coverage, preferably a monolayer at the surface of an electrode with deposited gold nanoparticles (AuNPs), is a critical factor for constructing a biosensor that can push temporal resolution from the sub-second limit of conventional probes down to the millisecond time scale [26]. Other biosensors for in vivo analysis have been based on the attachment of enzyme multilayers to improve sensitivity and additionally a protective film, such as nafion®, or a size exclusion membrane to meet the requirements needed for selectivity for in vivo analysis [27]. A thicker layer of enzyme coating affects the sensor temporal resolution since the reporter molecule that is produced after the enzymatic catalysis reaction has a longer distance to travel to be detected by the electrode compared to the minimal distance needed to diffuse when the enzyme coating is limited to a monolayer. On the other hand, more enzymes present at the sensor surface produce a larger amount of detectable product that enhances the sensor sensitivity. So basically, this comes down to a tradeoff between sensor sensitivity and temporal resolution [28]. Thus, depending on the specific sensor application, one or the other of these two parameters needs to be prioritized at sensor design and will determine what kind of enzyme surface interactions that needs to be optimized for enzyme immobilization. Upon attachment to a surface, enzymes can either maintain the macromolecular shape or to various degrees alter the tertiary structure. If any level of denaturation is induced, this can result in alterations of enzymatic activity and selectivity when enzyme binds to a surface, and therefore, it is important to characterize the enzyme when immobilized to the specific surface material that will be used in sensor fabrication. The characterization of immobilized enzymes is especially important for biosensors that require multiple sequential enzymes in order to produce an electro-active detectable reporter molecule, as the enzymatic activity in bulk might differ significantly for one or more of the enzymes when attached. Therefore the theoretical optimal ratio between enzymes may vary greatly when co-immobilized at the sensor surface and hence to achieve optimal sequential enzymatic activity, analysis should be performed after adsorption of various ratio of enzymes added to the surface [26].

To functionalize the electrode surface with nanoparticles, protocols that supply coatings with desired nanoparticle size and surface coverage are central to attain. In this work, we have optimized conditions for electrodeposition of AuNP at the surface of a glassy carbon (GC) electrode. To characterize enzyme adsorption at the AuNP surfaces of the electrodes, it is important to correlate the quantitative results of enzyme coating to the surface analysis of each individual electrode. Therefore, after AuNP deposition to perform a careful characterization of the nanoparticle size and population density at the sensor surface scanning electron microscopy imaging analysis is commonly used [23, 29]. The Compton group also developed an alternative method that electrochemically measures the gold surface at the modified electrode and by electrochemically stripping off the AuNPs, the average size and density of the AuNPs that had been deposited on the electrode surface was determined [29]. Previous methods for quantification of immobilized enzyme at a nanoparticle structured electrode surface, enzymes were labeled with a fluorescent tag before attaching to AuNPs at the surface of a carbon electrode and then by dissolving the AuNPs in KCN the freed enzymes in solution were quantified using fluorimetry [30, 31]. As characterizing the AuNPs size and density at the sensor surface first with SEM imaging and then by dissolution of AuNPs in KCN is time consuming and a toxic process, we therefore developed a new method that is significantly faster and safer to work with. In this new method, we combine the determination of the size and density of electrodeposited AuNPs at the surface of a GC electrode by an electrochemical stripping procedure. This was followed by quantification of fluorophore labeled enzymes that were immobilized to the AuNP coated GC electrode and subsequently released after the AuNP stripping procedure using fluorimetry, which directly provides information on the number of immobilized enzymes at the AuNP surface in a non-toxic method. The presented method greatly facilitates the characterization of enzyme-based AuNP structured electrochemical biosensors. Both with the goal of finding the optimized conditions needed for enzyme monolayer coverage for design of a sensor with high temporal resolution, as well as for identifying the amount of enzyme immobilized when several layers are desired as described above. This method will also work for quantifying several different enzymes immobilized simultaneously since each type of enzyme can be labeled with different fluorescent tags [31].

## Materials and methods

### Chemical reagents

AlexaFluor 488 protein labeling kit was purchased from Invitrogen (Carlsbad, CA). Glucose oxidase from *Aspergillus niger* (type VII), sodium phosphate dibasic, potassium phosphate monobasic, sodium chloride, sodium bicarbonate, sulfuric acid, copper sulfate, acetic acid, sodium acetate, and ferrocene methanol was purchased from Sigma-Aldrich (St. Louis, MO). All reagents used where of reagent grade and used as received. Deionized water (resistivity ≥ 18 MΩ cm) was used in all experiments.

### Electrochemical setup

Electrochemical measurements were performed using a three-electrode system with a computer-controlled 1000C Series Multi-Potentiostat from CH Instruments, USA. For all experiments a 3-mm in diameter GC electrode (CH Instruments, USA) was used as working electrode, a platinum electrode as auxiliary electrode and a saturated Ag/AgCl was used as reference electrode unless otherwise stated. All potentials are reported relative to the NHE electrode potential. Prior of use the GC electrode was polished with an alumina slurry (0.05 μm particles) according to the protocol provided from CH Instruments, USA. After polishing, the electrodes were sonicated in deionized water for 10 min in an ultrasonic bath and extensively rinsed in DI water. All electrodes were tested in 1 mM ferrocene methanol by performing cyclic voltammetry between 0 and 0.8 V at 0.1 V s ^−1^ and each voltammogram was evaluated in order to verify the electrodes were well-functioning prior to each experiment.

### AuNP functionalization at the GC electrode surface

Electrodes were functionalized with AuNPs by using an electrochemical deposition protocol similar to Finot et al. [25] with minor alterations in the gold chloride concentration and deposition time used in order to optimize AuNP size and electrode coverage. Briefly, the AuNPs were electrodeposited onto the GC electrode surface using a 1-mM HAuCl_4_ solution in 500 mM H_2_SO_4_. Electrodeposition was performed by applying a potential of + 1.4 V for 10 s followed by a potential of − 0.4 V for 24 s. After deposition, the electrode was extensively rinsed with deionized water.

### Electrochemical measurements of AuNP density and size at the electrode surface

An electrochemical linear sweep method adapted by Finot et al. was used in these experiments for the determination of the total surface area of the AuNPs coating the electrode surface [25]. Briefly, a constant potential of + 1.7 V (vs. NHE) was applied to the electrode surface placed in a 500-mM H_2_SO_4_ solution and was held constant for 5 s before sweeping the potential at 0.1 Vs^−1^ down to + 0.8 V. _._The resulting reduction peak at approximately + 1.1 V was recorded and integrated to determine the associated charge detected from the induced redox reaction at the AuNP surface. A variety of coefficient values for relating the total charge transfer from the reduction of a monolayer of oxides at the gold electrode surface to the total gold surface area at the electrode have been reported by different research groups, e.g., a constant corresponding to 543 μC cm^−2^ as proposed by Habrioux, A., et al., [32] 400 μC cm^−2^ proposed by Trasatti, S. and O. Petrii, [33], and 450 μC cm^−2^ was suggested by Tan. et al. [34]. In this study, a factor of 489 μC cm^−2^ as determined by Finot et al. was used in this work as a similar method for electrodeposition of the AuNPs to the electrode surface was applied [25]. The accuracy of using this factor was confirmed by the SEM image analysis of the AuNP modified electrodes. A Cu/CuSO_4_ reference electrode was used instead of an Ag/AgCl to avoid chloride contamination since the presence of chloride ions will dissolve the deposited gold when high anodic potentials are applied to the electrode surface during the electrochemical AuNP surface analysis [35]. The AuNPs at the GC electrode surface was electrochemically stripped off using a method described by Wang et al. [29] with minor alterations to adjust the conditions needed for enzyme dissolution. Here the AuNPs were stripped off into a 100-mM hydrochloric acid solution or in the case for enzyme dissolution the AuNPs were stripped off into a 100-mM acetate buffer solution containing 100 mM sodium chloride. To ensure all gold to be dissolved from the electrode surface, 6 sequential voltammetry cycles ranging from + 0.9 V to + 1.5 V (vs. NHE) with a scan rate of 0.1 Vs^−1^ was applied to the electrode surface, where generally all the gold was observed to be stripped off after the first cycle. The resulting oxidation peak observed at approximately + 1.3 V was integrated and together with the gold surface area as determined from the linear sweep analysis, was used for calculation of the average AuNP radius and the number of AuNPs electrodeposited at the electrode surface. After the AuNP stripping analysis, the sweep was repeated when electrodes were placed in a 500-mM H_2_SO_4_ solution, as described above, to ensure that all gold was eliminated from the electrode surface. To verify the accuracy in the electrochemical methods to determine AuNP size and surface coverage, SEM imaging of the AuNP coated electrode tip surfaces was performed using a LEO Ultra 55 FEG (Carl Zeiss, Germany) equipped with a field emission gun and an electron backscattered diffraction detector. The AuNP deposited electrodes were attached and grounded to an electrode holder using a tungsten wire for limiting the effect of charging during imaging. SEM image analysis of AuNP size and coverage was determined using the software *Image J*. (National Institutes of Health, USA).

### Enzyme labeling with a fluorescent tag

For enzyme labeling with the fluorophore AlexaFluor488, a labeling kit protocol provided by Invitrogen (ThermoFisher Scientific) was used. Briefly, the enzyme, GOx, was first suspended in a phosphate-buffered saline (pH 7.4) at a 2 mg ml^−1^ concentration followed by raising the pH of the solution using sodium bicarbonate (pH 8.3). The enzyme solution was incubated with the fluorescent dye over night at 4 °C after an initial incubation of 1 h in room temperature with continuous stirring. The labeled enzyme was separated from excess dye using a size exclusion column according to the labeling protocol. To determine the average number of fluorophores attached per enzyme and the final enzyme concentration after the labeling procedure, the enzyme solution was analyzed using a Cary 4000 UV-Vis spectrophotometer (Agilent Technologies Inc., USA) according to the protocol provided by Invitrogen.

### Immobilization of enzymes at the AuNP coated electrode surface

To immobilize enzymes at the AuNP coated surface of the GC electrode, the tip of each electrode was immersed into a 300 μL of a 10-mM sodium phosphate buffer solution containing fluorescently labeled GOx (0.2 mg mL^−1^), pH 7.4 for 3 h incubation time at room temperature. In this process enzymes attach through self-adsorption and after the enzyme coating process, the tip of each electrode was washed extensively with deionized water.

### Quantification of the number of enzymes covering the AuNP surface of an electrode

The enzyme coated electrodes were immersed in to 200 μL of 100 mM acetate buffer containing 100 mM NaCl at pH 4. A chlorinated silver wire and a silver wire were used as reference and auxiliary electrode respectively in order to fit in the small volume used for the anodic stripping analysis. The chlorinated silver wire reference electrode was calibrated towards an Ag/AgCl saturated reference electrode prior to use. The AuNPs was electrochemically stripped off as described above, resulting in both dissolution of AuNP and freeing the AuNP adsorbed enzymes into solution. The electrochemical stripping solution containing the fluorescently labeled GOx was quantified using a Cary Eclipse fluorescence spectrophotometer (Agilent Technologies Inc., USA) and 494/519 nm as the excitation/emission wavelengths. A calibration curve for the labeled enzyme in acetate buffer (100 mM, pH 4) with 100 mM NaCl was performed for each new batch of labeled enzyme.

## Results and discussion

In this work, we have developed a new fast, facile and non-toxic analytical method to characterize an enzyme-based AuNP coated biosensor with respect to the size and density of AuNPs at a carbon electrode surface together with quantification of the number of enzymes immobilized at the AuNP coating of the electrode. Since high curvature surface support as achieved with NPs has been shown beneficial for maintaining enzyme tertiary structure and thereby retaining enzyme activity upon immobilization, we here electrodeposited AuNP as enzyme support at the surface of a carbon electrode [12, 13]. We here characterize sensors for enzyme coverage and total electrode surface area. Sensors optimized for achieving monolayer coverage of enzyme adsorbed onto the AuNP coated electrode surface have shown to be favorable towards achieving both high sensor sensitivity and temporal resolution [26]. Therefore, the coverage of the AuNPs at the electrode surface need to be optimized since too few NPs will lead to a poor enzyme loading and too many will lead to the formation of a gold film rather than discrete AuNPs, which results in losing the advantage with high curvature surface for retaining enzyme activity after immobilization. Using an electrochemical method for direct determination of the average AuNP size, the number of AuNP and the total AuNP surface area at the sensor surface replaces the need for scanning electron microscopy imaging analysis of each electrode for characterizing the nanostructure size and density at the surface, which is a time-consuming process that requires sampling of a number of representative images at the surface of each electrode for image analysis. The great advantage of the electrochemical approach is that the number of enzyme immobilized at the AuNP support at the sensor surfaces can be determined directly and related to each individual electrode. Due to the nature of electrochemical deposition of NPs at an electrode surface resulting in a variability in density and NP size at each individual electrode, this direct analysis method enables a direct characterization of each nanostructured electrode and facilitates determination of for instance enzyme loading at electrode NP surface coatings where NP size or density might vary.

### Characterizing AuNPs size and coverage at the electrode surface

**Fig. 1:**
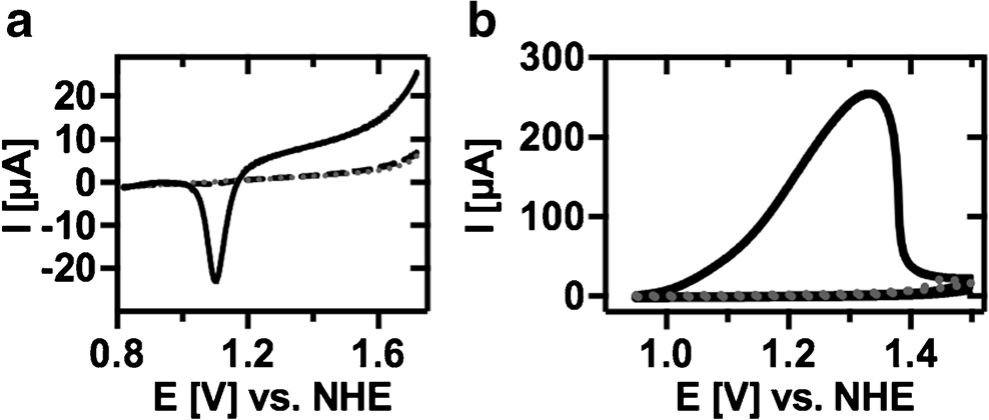
Voltammograms of (**a**) the measured total AuNP surface area by performing a linear potential sweep at the electrode surface when placed in 500 mM H_2_SO_4_ after AuNP modification (solid)_,_ after anodic stripping (dotted) and at a bare GC electrode (dashed). (**b**) an anodic stripping of AuNPs (solid) at the electrode surface when placed in 100 mM Acetate buffer, 100 mM NaCl, pH 4 and at an unmodified bare GC electrode (dashed). Scan rate 0.1 V s^−1^

To characterize the AuNP coating at the electrode surface after electrodeposition, the total AuNP surface area was first measured using a linear sweep voltammetry method developed by Finot et al. [25]. This was accomplished by applying a 5-s constant potential of + 1.7 V vs. a NHE reference to the AuNP coated electrode surface in an acidic environment, inducing an electro-oxidation reaction to take place at the AuNP surface, and was followed by sweeping the potential down to + 0.8 V, resulting in a reduction reaction of the gold oxide species as shown by the recorded current peak displayed in Fig. 1a. By integrating the resulting cathodic peak present at approximately + 1.1 V and using a coefficient value of 489 μC/cm^2^, corresponding to the charge required to reduce a monolayer of divalent oxygen on a polycrystalline gold surface, the total AuNP surface was determined [25].

By electrochemically stripping off the AuNPs deposited at the electrode surface by performing cyclic voltammetry, the charge from the oxidation peak, (Fig. 1b), can together with the electrochemically measured total AuNP surface area be used to calculate the average AuNP size and number of AuNP deposited at the GC electrode surface using Eqs. (1–3) below [29]. In these experiments, a stripping method by Wang et al. [29] with minor alterations was used. It was noted that no reduction peak was observed from either the bare GC electrode, or the modified electrode after anodic stripping, indicating that all AuNPs were dissolved from the electrode surface after the stripping. The charge *Q* as determined from the integrated oxidation peak during the anodic stripping of the AuNPs can be expressed as:

1 $$ Q=1.9\times e\times \frac{\frac{x}{3}\pi {r}^3{\rho}_{Au}}{M_{Au}}\times {N}_A\times N $$

where *N* is the number of AuNPs, *r* is the average radius of AuNPs, *e* is the electronic charge, *M*_*Au*_ is the atomic mass of gold, *x* = 2 (for hemispherical NPs, assumed on the electrode surface), 1.9 is the average number of electrons transferred per gold atom, which is quantitatively related to the charge of the stripping. *N*_*A*_ is Avogadro’s number and *ρ*_*A*u_ is the density of gold. From the linear potential sweep in sulfuric acid, the total surface area *S* of AuNPs is measured and is related to the size and number of AuNP at the surface according to:

2 $$ S= x\pi {r}^2\times N $$

which means that the AuNP radius can be calculated by:

3 $$ r=\frac{3{M}_{Au}}{1.9e{\rho}_{Au}{N}_A}\times \frac{Q}{S} $$

Gold dissolution will occur at anodic potentials high enough for oxygen evolution, but will greatly be enhanced in a concentration dependent manner in the presence of halide ions such as chloride. The dissolution process of gold is also depending on the proton concentration in the solution where a low pH in the presence of chloride ions increases the dissolution [35, 36]. During the anodic stripping, the gold will be oxidized [37–39], and the outcome will result in the species from the following equilibrium:

4 $$ Au+4{Cl}^{-}\rightleftarrows {AuCl}_4^{-}+3{e}^{-} $$

The auric chloride can further be reduced according to the comproportionation reaction:

5 $$ Au{Cl}_4^{-}+2 Au+2{Cl}^{-}\rightleftarrows 3 Au{Cl}_2^{-} $$

**Fig. 2:**
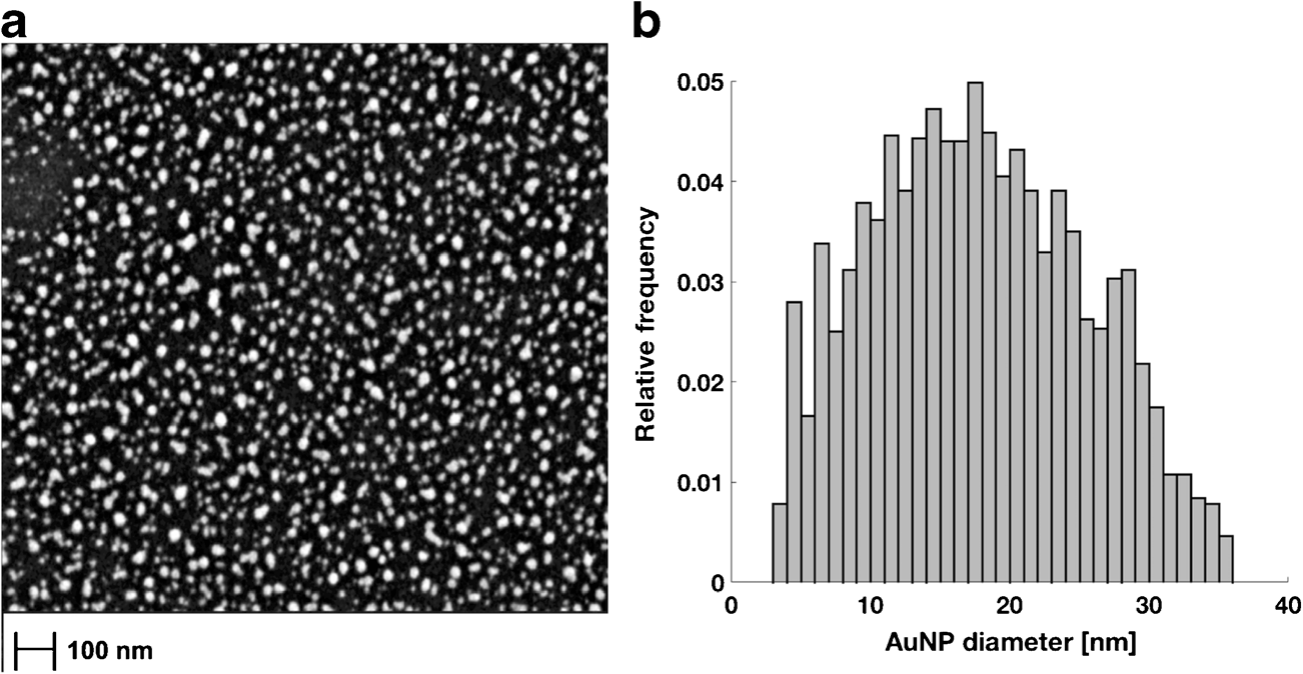
(**a**) A representative SEM image of AuNPs electrodeposited at the surface of a GC electrode. (**b**) A histogram of the diameter of individual AuNPs (*n* = 3429) collected from four SEM images taken randomly at the surface of one individual AuNP coated GC electrode as determined by SEM image analysis.. Adjusted R-square to a Gaussian fit of the AuNP population corresponds to 0.9

Using this electrochemical analysis methodology to characterize individual electrodes (*n* = 69) in terms of AuNP size and surface coverage, an average diameter of 17 ± 6 nm and a surface geometric area coverage of 41 ± 12% were determined. These results were confirmed by characterization of individual electrode surfaces using SEM image analysis (*n* = 3) as shown in Fig. 2 and result in an average AuNP diameter of 20 ± 8 nm and a surface coverage of 27 ± 7% which verifies the accuracy of this electrochemical analysis method to characterize the nanoparticle structured electrode surface.

### Counting the number of enzymes immobilized onto the AuNP surface

**Fig. 3:**
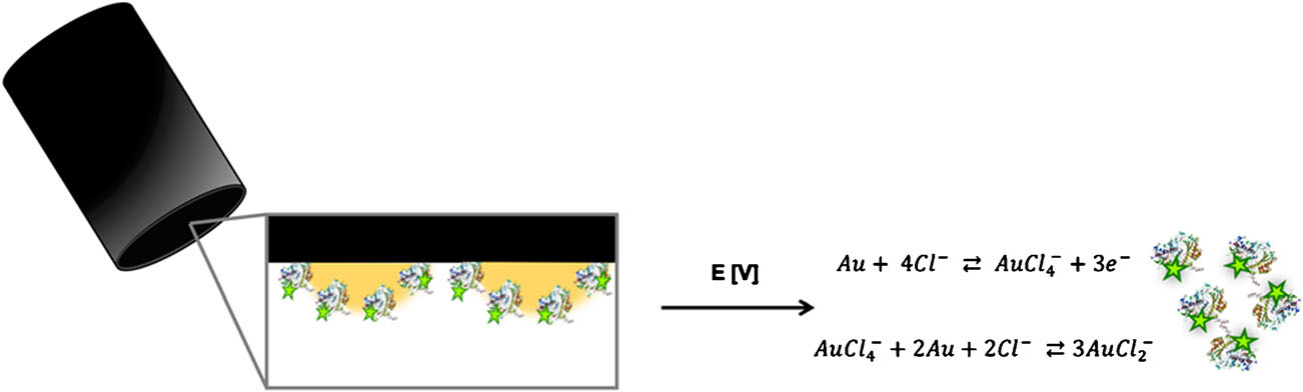
Fluorescently labeled GOx immobilized onto the AuNP surface coating of a GC electrode. By immersing the electrode into an acidic solution containing chloride ions and cycling a potential between + 0.9 and + 1.6 V the AuNPs electrochemically dissolves and immobilized GOx are released into solution. The fluorescent tag is indicated as a star at the enzyme

To quantify the number of enzymes immobilized onto the AuNPs at the GC electrode surface, enzymes were labeled with a fluorescent tag. In this work, we labeled the enzyme glucose oxidase (GOx) with Alexa-488 before immobilization onto the electrodeposited AuNPs at the electrode surface by physical adsorption. During the anodic stripping, the AuNPs were electrochemically dissolved and the labeled enzymes were released into the stripping buffer as schematically described in Fig. 3. The dissolved enzymes from the anodic stripping was directly quantified using fluorimetry.

In the original method developed by Wang et al. [29], HCl, an electrolyte solution, was used for the anodic stripping off the AuNPs. Due to that the fluorescence dye Alexa 488 used for labeling the enzyme is stable in solution with a pH interval between pH = 4 and pH = 10, the stripping solution was replaced with a 100-mM acetate buffer with pH 4 and containing 100 mM NaCl. Performing this exchange of anodic stripping solution did not affect the quantitative results in these experiments determining the size or density of the AuNPs at the electrode surface as presented by the summarized results in Table 1 comparing different stripping conditions. These results also demonstrate that covering the AuNPs with GOx before anodic stripping did not significantly affect the quantitative results.

**Table 1:** Comparison of AuNP size, total number of AuNP at the electrode surface, total AuNP surface area and surface coverage of electrodeposited AuNPs at the GC electrode surface when using three different conditions for the anodic stripping

Solution	AuNP diameter (nm)	Number of AuNP (10^9^)	AuNP surface area (10^−2^) cm^2^	AuNP coverage %
HCl	19 ± 3	10 ± 4	5.2 ± 1.9	36 ± 13
Ac	17 ± 7	19 ± 11	6.5 ± 1.4	46 ± 10
Ac + GOx	15 ± 7	24 ± 11	4.3 ± 2.2	41 ± 10

Stripping was performed in HCl solution (HCl) (*n* = 25), acetate buffer (Ac) (*n* = 18) and in acetate buffer with enzymes immobilized at the surface (Ac + GOx) (*n* = 26). Results are presented as average values together with standard deviation.

GOx is an elongated globular protein consisting of two subunits with a dimension of approximately 8 nm along the major axis and 7 nm by the minor axis [39, 40]. As shown previously in literature, GOx have a tendency to flatten out at surfaces after adsorption depending on the surface properties of the immobilization support. This might also be affected by the concentration of enzyme at the surface, where crowding will hinder unfolding of the enzyme due to the lack of space to spread out [41–43]. Hence, even though the dimension of GOx is known, the number of enzyme that can be expected to fit onto a surface might deviate from theoretical calculation depending on the extent to which the tertiary structure is disrupted upon adsorption. At a surface where the unfolding of the protein structure is not initiated, the GOx footprint when adsorbing to a surface is estimated to range from 21 to 67 nm^2^ depending on if the enzyme attaches by the short or the long side to the solid support. However, at a flat gold surface the footprint was observed to increase up to approximately 290 nm^2^ when the enzyme totally collapsed onto the surface [44]. Studies of the enzyme interaction with surfaces of other materials, with different topology and at different experimental conditions, shape changes of less extent were observed resulting in a footprints reported to vary in the range of about 70–144 nm^2^ when GOx adsorbed to these surfaces [41, 42].

To calculate the average number of enzymes attached per AuNP at the surface of each individual GC electrode (*n* = 6), the quantification of immobilized GOx at the AuNP surface of the electrode was directly related to the average AuNP size as determined electrochemically. Although the AuNP display a heterogeneous size distribution and a variability in particle density between individual electrodes, the population of AuNP at each individual electrode surface displayed a Gaussian distribution in terms of AuNP size and the resulting total AuNP surface area was rather constant. Hence, using the method for electrodeposition of AuNP offers roughly the same total available surface area for enzymes to bind and display an average diameter of AuNP corresponding to 14 ± 6 nm at the electrode surface.

**Fig. 4:**
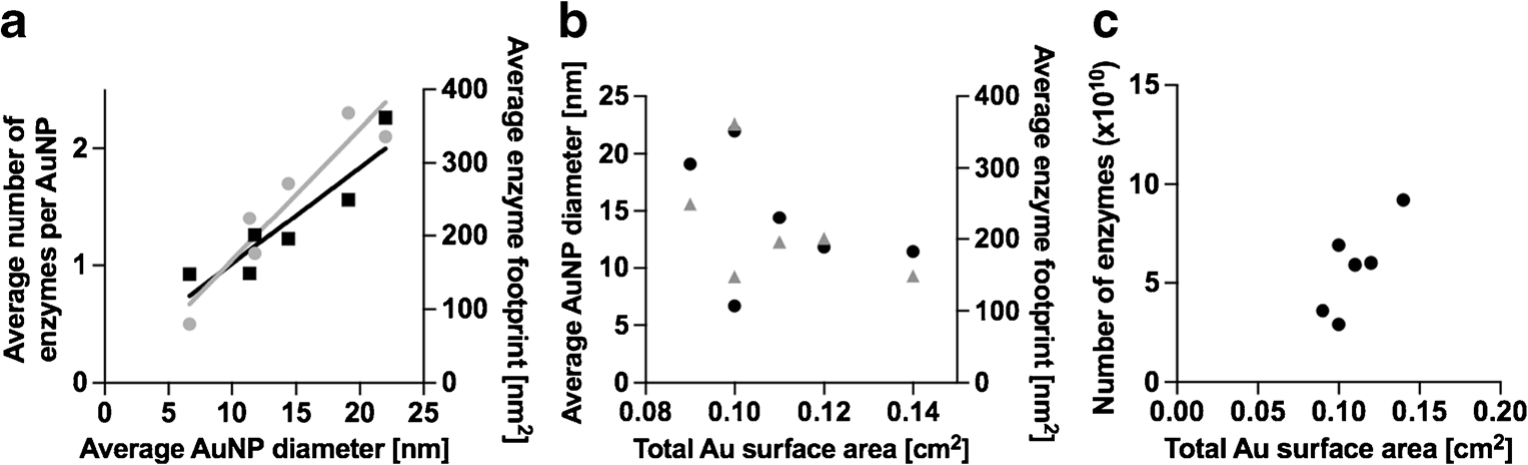
(**a**) The average number of immobilized enzymes per AuNP as determined by fluorimetry after AuNP stripping at the electrode was used to calculate the average enzyme footprint at the AuNP surface and plotted against the average size of electrodeposited AuNP as determined by electrochemical analysis. Average number of enzymes per AuNP is indicated as spheres (*R* ^2^ = 0.88) and average enzyme footprint is indicated as squares (*R* ^2^ = 0.83). (**b**) The average size of AuNP as determined by electrochemical analysis and the corresponding calculated average enzyme footprint size as determined at each individual characterized electrode and plotted versus the average AuNP diameter of individual electrodes as determined by electrochemical analysis. Enzyme footprint is indicated as triangles and AuNP diameter as spheres. (**c**) The total number of enzymes immobilized at the AuNP surface as determined by fluorimetry versus the total AuNP surface area at the AuNP modified electrode

Considering that these nanoparticles have a geometrical shape of a half sphere after electrodeposition at the electrode surface results in an average surface area of approximately 300 nm^2^. Therefore, theoretically if the enzymes would adsorb with the short side to the AuNP surface without experiencing any shape changes or steric hindrances by other enzymes adsorbed at the surface, a maximum of 15 GOx can densely pack onto a 300-nm^2^ surface. If the enzyme adsorbs by the long side, up to 4 enzymes can fit onto the AuNP surface. However, if steric hindrance affects the packing or the enzyme experience flattening at the AuNP surface much fewer enzymes can be expected. By correlating the quantification results of immobilized enzymes as determined by fluorescence to the number of AuNP deposited at the electrode surface and also the total AuNP surface area as characterized by electrochemical analysis and SEM image analysis, the average number of enzymes per AuNP correspond to 1.5 ± 0.7. This offers each enzyme an average footprint area of 200 nm^2^. Although the average size of the AuNP population between electrodes display some variability, the consistency in total AuNP surface area offers a constant total available surface area for enzymes to bind. This is supported by the linear dependence of the average number of enzymes immobilized per AuNP to the average size of AuNP electrodeposited at the electrode surface and the total AuNP surface area, as shown in Fig. 4a, b. As displayed in Fig. 4c, the total number of enzymes that bind is related to the total AuNP surface area available (Fig. 4c). However, the enzyme footprint area shows variability with the AuNP size, where at smaller particles with higher curvature, resulting in a smaller enzyme footprint and may provide a scenario for the enzyme to better retain its 3D structure. These quantitative results also point to that the GOx concentration used for enzyme immobilization gives rise to roughly a monolayer coverage at the AuNP modified electrode surface. Additionally, by assuming that the enzymes are fully covering the AuNP surface area, and considering the nature of GOx tendencies to flatten during surface adsorption, the enzyme footprint size displayed here indicates that moderate shape changes of the enzyme occurs upon adsorption to the AuNP support at the electrode surface.

As the interaction with a biomolecule to a AuNP surface is unique to the molecular properties of the biological component and is also related to the size of the AuNP, it is impossible to predict tendencies for macromolecular denaturation upon immobilization to a surface and therefore it is often a lot to gain in biosensor design and fabrication if studying the optimal conditions for the bioconjugation to ensure the bioactivity is ideal. In fabrication of enzyme based electrochemical sensors, the AuNP size affects sensitivity where for instance smaller AuNPs also possess increased electron density making them more electroactive and thereby more efficient in detecting the enzymatic product hydrogen peroxide. Hence, this electrochemical-based method offers a simple non-toxic way for quantitative analysis of fluorescently labeled immobilized biological component at the surface of biosensors based on gold bioconjugates. This analysis method therefore also should apply to many common biorecognition elements such as enzymes, antibodies, receptors, DNA or RNA bound to the sensor surface. In optimization of AuNP size used for immobilization of the biological component, this method can greatly facilitate this work and can also be used for quantification of co-immobilized biological components at a AuNP coated sensor surface. An example of this is for instance in finding the optimal conditions for immobilization of sequential enzymes that result in an optimum enzyme ratio to achieve prime sequential enzymatic reaction and can be performed by labeling each immobilized enzyme with an individual fluorescent tag.

## Conclusions

Here we present a new facile, fast and non-toxic method to quantify the number of GOx enzyme that is immobilized to the surface of a AuNPs coating of a GC electrode. This method is based on combining an electrochemical analysis method, SEM image analysis and fluorimetry. An electrochemical method was used to characterize the density and size of electrodeposited AuNP at a GC electrode sensor surface and SEM image analysis was used to verify the electrochemical characterization of the AuNP structured sensor surface. After characterization of each individual AuNP coated GC electrode, these surfaces were coated with fluorescently labeled enzyme that attach through adsorption. Quantification of the immobilized enzyme was achieved by fluorescence measurement of the fluorophore-labeled enzymes after they were freed in solution by dissolving the AuNP support to which the enzymes were bound, using an electrochemical stripping technique. We show that by counting the enzymes bound to the AuNP surface we identified that the immobilization conditions used here result in roughly a monolayer coverage of enzymes at the electrode surface. And an enzyme footprint that indicate that depending on the level of enzyme using the available surface area to bind and spread upon adsorption, the enzyme at these conditions seem to be experiencing moderate to major shape changes. This analytical new method is of great importance for characterizing and optimizing the conditions needed to fabricate enzyme-based electrochemical biosensors, since an understanding of the surface processes for immobilizing the enzyme directly affects the sensor sensitivity and hence allows to determine the retained enzymatic activity after immobilization to the electrode surface. In addition, by limiting the enzyme coating to monolayer coverage is a key factor for further optimization of biosensors when it comes to achieving a high temporal resolution for detection of non-electroactive analytes by these biosensor probes. In summary, this method offers the possibility to characterize the sensor surface in terms of AuNP size and coverage and to perform quantitative analysis of different biological components that are used in bioconjugation when fabricating a biosensor. We show that fluorescent labeling allows for fluorescent quantitative analysis of the immobilized biomolecules, but depending on the properties of the biomolecules other direct quantitative analysis methods might be considered to determine the number of biomolecules bound to the surface before their support is electrochemically stripped off at the sensor surface. Therefore, we believe that this methodology might be very useful for optimizing the conditions when designing and fabricating many different kinds of biosensors where a biological recognition element is immobilized at an AuNP support.
